# Effect of the 2018 Japan floods and COVID-19 pandemic on cognitive decline among atomic bomb survivors in Hiroshima, Japan: a retrospective cohort study

**DOI:** 10.1007/s40520-025-03054-z

**Published:** 2025-05-16

**Authors:** Shuhei Yoshida, Daisuke Miyamori, Masanori Ito

**Affiliations:** Department of General Internal Medicine, Hiroshima University Hospital, 1-2-3 Kasumi, Minami- ku, Hiroshima-shi, Hiroshima-ken 734-8551 Japan

**Keywords:** Flood, COVID-19 pandemic, Cognitive function, Atomic bomb survivor, Welfare recipient

## Abstract

**Background:**

Atomic bomb survivors (ABSs) in Hiroshima are facing climate change-related natural disasters and emerging infectious diseases. The cognitive function of aging ABSs is vulnerable to the inevitable environmental changes caused by the 2018 Japan floods and COVID-19 pandemic.

**Aims:**

This study examined the effects of these two disastrous events on cognitive function.

**Methods:**

This retrospective cohort study included all verified individuals utilizing Long-Term Care Insurance services in Hiroshima Prefecture. The observation period was from January 2018 to December 2022. The participants were divided into three groups: ABSs, welfare recipients (WRs), and others. The objective variable was moderate or severe cognitive deterioration. We performed a difference-in-differences analysis using logistic regression models to investigate the effect of the two disastrous events on cognitive function compared with the effect of the other groups.

**Results:**

There were 184,252 participants, including 16,306 ABSs (8.8%) and 8,509 WRs (4.6%). The difference-in-differences analysis showed no statistically significant effect of the 2018 Japan floods. The analysis also revealed that moderate cognitive decline among ABSs and WRs decreased during the COVID-19 pandemic (2020, 2021, and 2022). Moreover, severe cognitive decline among ABSs decreased after the onset of the COVID-19 pandemic (2020 and 2021).

**Discussion:**

Although many older adults experienced cognitive exacerbations during the COVID-19 pandemic, ABSs had a lower risk of cognitive decline than those of non-WRs. However, no significant changes were observed during the 2018 Japan floods.

**Conclusions:**

ABSs had a reduced risk of cognitive decline during the pandemic compared with that of the other groups.

## Introduction

Hiroshima City, located in western Hiroshima Prefecture, was hit by an atomic bomb in 1945. Survivors who suffered damage from the atomic bomb were certified as atomic bomb survivors (ABSs), or “*Hibakusha*,” if they met the criteria set by the Japanese government [1]. The health problems of ABSs include an increased risk of various types of cancer as well as psychological conditions, such as depression and posttraumatic stress disorder [2]. The effects of the atomic bombing have been long-term. To address these health concerns, the Japanese government has implemented comprehensive support schemes for certified ABSs, including waivers for medical and long-term care copayments.

In addition to these support schemes, ABS status may have contributed to societal labeling, influencing their experiences as a distinct group [3, 4]. Although ABSs do not necessarily experience adverse life outcomes in terms of average marital status or education, they have faced significant disadvantages in several areas, including marital prospects, employment, mental health, and future expectations [5]. ABSs in Hiroshima tend to have higher levels of trust in others than those of individuals born in other regions of Japan [6]. This finding suggests that ABSs may exhibit distinct social responses to events unrelated to the atomic bombing.

In recent years, Hiroshima Prefecture has been severely affected by climate change-related natural disasters and emerging infectious diseases. One of the most substantial natural disasters was the 2018 Japan flood, which caused 237 deaths, eight missing persons, 433 injuries, and the destruction of 6,767 houses [7]. In addition to its direct impact, the disaster worsened cognitive function among older adults, contributed to an increase in certain diseases, and led to rising healthcare costs [8–11]. Two years later, the COVID-19 pandemic emerged, affecting Hiroshima as well as other regions and countries [12]. Beyond the direct health risks posed by the virus, disease-related social isolation became a major factor in the acceleration of cognitive decline and increased mortality risk from non-COVID-related causes [13, 14].

As the ABS population continues to age, they are increasingly vulnerable to the effects of natural disasters and pandemics. These events have resulted in significant environmental changes. Evacuation measures and social isolation, which disrupt communication within older adults’ communities, may exacerbate cognitive decline. Moreover, these events have increased care needs, resulting in lasting effects even after the crises ended [15, 16]. However, no studies have examined the cognitive function of ABSs during such events. Notably, research has found that radiation exposure does not significantly influence the incidence of dementia or its subtypes [17]. Therefore, if ABSs exhibit a different trajectory of cognitive function compared with that of others, this could indicate the influence of bio-psycho-social factors unrelated to radiation exposure. Identifying these differences could inform strategies for preventing cognitive decline during future catastrophic events. Moreover, such findings would provide further evidence of the long-term societal impact of labeling people as members of a special population.

## Method

### Study design

This study was a retrospective cohort study.

### Long-term care insurance (LTCI) system in Japan

The LTCI, established in 2000, is a mandatory public program that provides benefits for the long-term care of older adults. All persons aged ≥ 65 years can use the LTCI service with care-level certification. The LTCI system, which is administered by municipalities, covers various services, such as residential facilities, home visit care, and short-term residential care. To use LTCI services, individuals must first apply for care-level certification. This involves an investigation of their physical and mental condition by care-related professionals and doctors. The Certification Committee of Needed Long-Term Care determines the level of care needed, which is divided into seven levels (support requirement levels 1–2 and care requirement levels 1–5). Service users pay a copayment of 10 to 30% of the service cost, with the remaining costs covered by the LTCI budget. Recertification is conducted when the certification period ends or when the user needs care services beyond the permitted certified care level because of disease progression or a decline in activities of daily living or cognitive function.

### Data on LTCI users

This study was conducted using certification data for long-term care and LTCI claims (approval no. 0905-1). The Ministry of Health, Labour and Welfare (MHLW) maintains the Japanese Long-Term Care Database. This database contains digitized claims for LTCI services, which are summarized monthly, and includes detailed information on the services used by each individual. The MHLW has been granting research institutions access to datasets derived from this database since 2018, following approval by expert councils.

### Setting

The study was conducted in Hiroshima Prefecture, Japan. Hiroshima and Nagasaki Prefectures have the highest concentration of ABSs. Hiroshima Prefecture was chosen as the setting because it has experienced both of the aforementioned disasters: the 2018 Japan floods and COVID-19 pandemic.

### Participants

The participants were all LTCI users who received care-level certification in Hiroshima Prefecture from January 2018 to December 2022. The observation period was from January 2016 to December 2022. When an ABS uses LTCI services, the amount equivalent to the out-of-pocket costs is exempted. Because the public expenditure numbers of ABSs are recorded in LTCI receipts, we were able to identify ABSs among LTCI users. We also identified welfare recipients (WRs), who were exempt from copayment, using public expenditure numbers in LTCI receipts to compare them with ABSs.

### Variables

Cognitive decline was used as the outcome variable. A doctor’s written opinion, which included a Dementia Symptomatology Assessment (DSA), was used to certify LTCI care levels. The DSA is a nationally standardized dementia scale designed to evaluate the level of independence in cognitive functions [18]. The care needs certification examination employs the same DSA scale. Although these investigations were conducted independently, their results demonstrated a strong correlation [18]. The DSA also has a high inter-rater reliability [19]. The DSA level exhibited a high correlation with the Mini-Mental State Examination, with level I being equivalent to 0.5 points on the Clinical Dementia Rating [20, 21]. We adopted the first deterioration to DSA level 3a or above and deterioration to DSA level M as the cutoff points. DSA level 3a is defined as “Symptoms, behaviors, and difficulties in communication that sometimes interfere with daily life and require nursing care.” This corresponds to the level at which a person is judged unable to live independently. We labeled DSA level 3a or higher as “moderate cognitive decline.” DSA level M is defined as “Significant psychiatric symptoms, problematic behavior, or serious physical illness requiring specialist medical care.” This is the highest level of DSA and is labeled as “severe cognitive decline.”

The primary exposure variable was ABS status. In addition, we considered WR status as an additional exposure variable. We used the public expense payer number (*Kouhi-Futansha-Bangou*) to identify LTCI users as ABSs or WRs. Users with payer number 19 were classified as ABSs, and those with payer number 12 were classified as WRs. Occasionally, an ABS was also a WR. In this case, we classified such individuals as ABSs rather than WRs because ABS status was prioritized for subsidized copayments. Age classification and sex were used as covariates.

### Statistical analysis

We used the chi-square test to compare dichotomous and categorical variables among ABSs, WRs, and others. To confirm the increase in cognitive decline after the start of the COVID-19 pandemic, we conducted a residual analysis to compare the observed and expected values derived from the care-level certification span. The duration of care-level certification is usually approximately 1 year. A short care-level certification span indicates a destabilized condition for LTCI users. If the certification span tended to shorten after the COVID-19 pandemic, cognitive function may have become more exacerbated because of this destabilization.

We performed a difference-in-differences (DID) analysis using logistic regression models. Because this observational study could not include randomized study participants, it was difficult to match the participant characteristics, including ABS status, WR status, other covariates, and unmeasured confounding factors, among the three groups (ABS/WR/Others). Therefore, it was necessary to address selection bias. Moreover, in a before-and-after study without a control group, it was necessary to adjust for natural historical trends over time. A DID analysis addresses these two biases if the time-series data of the exposure and control groups meet the common trend assumption before an event. The logistic regression models adopted cognitive decline as a dependent variable and the interaction term between ABS status and year, between WR status and year, ABS status, WR status, year, age classification, and sex as explanatory variables. Cluster-robust standard errors were calculated to account for repeated measurements among individuals. The first event examined was the 2018 Japan floods. Therefore, when the interaction terms between ABS status and years 2016 and 2017 and between WR status and years 2016 and 2017 were not significant (*p* > 0.05), the common trend assumption was considered satisfied. After confirming this assumption, we performed a DID analysis for the period 2018–2022. The frequency of outcome occurrences was less than 10%, and the results were presented as odds ratios. When the outcome variable indicated deterioration to moderate cognitive decline, participants with DSA levels of 3a or higher from the beginning of the observation period were excluded from the DID analysis. Similarly, when determining the progression to severe cognitive decline, participants with severe cognitive decline from the beginning of the observation period were excluded from the DID analysis.

All statistical analyses were performed using STATA/SE, version 18 (StataCorp, 2023).

## Results

Table 1 shows the characteristics of participants. The total number of participants was 184,252, including 16,306 ABSs (8.8%) and 8,509 WRs (4.6%). Age category and sex were statistically significant according to the chi-square test (*p* < 0.001). The number of participants with moderate deterioration in cognitive function from the beginning of the observation period was statistically significant, ranging from 1.8 to 2.9% in each group. The proportion of participants with severely deteriorated cognitive function before the observation period was 0.2% in the ABS group and 0.3% in the WR group. There was no significant difference between the groups (*p* = 0.627). Deterioration to moderate cognitive function during the observation period was observed in 7,092 (43.5%) ABSs, 2,938 (34.5%) WRs, and 57,355 (36.0%) others (*p* < 0.001). Deterioration to severe cognitive function was 1,060 (6.5%) in the ABS group, 799 (12.5%) in the WR group, and 17,577 (11.0%) in the other groups (*p* < 0.001).

**Table 1:** Characteristics of participants

		Atomic bomb survivor		Welfare recipient		Others		P value
		*n* = 16,306	8.8%	*n* = 8,509	4.6%	*n* = 159,437	86.5%	
		N	%	N	%	N	%	
Age category	65–74	944	5.8	3,908	45.9	26,813	16.8	*p* < 0.001
	75–84	6,125	37.6	3,488	41.0	73,499	46.1	
	85–94	8,760	53.7	1,054	12.4	55,722	35.0	
	95-	477	2.9	59	0.7	3,453	2.2	
Sex	Male	4,772	29.3	3,320	39.0	52,393	33.0	*p* < 0.001
	Female	11,534	70.7	5,189	61.0	107,033	67.1	
Baseline cognitive function	Moderate	462	2.9	170	2.1	2,792	1.8	*p* < 0.001
	Severe	33	0.2	22	0.3	338	0.2	*p* = 0.627
Deterioration of cognitive function during observation period	Moderate	7,092	43.5	2,938	34.5	57,355	36.0	*p* < 0.001
	Severe	1,060	6.5	799	12.5	17,577	11.0	*P* < 0.001

P value: Chi-square test

**Fig. 1:**
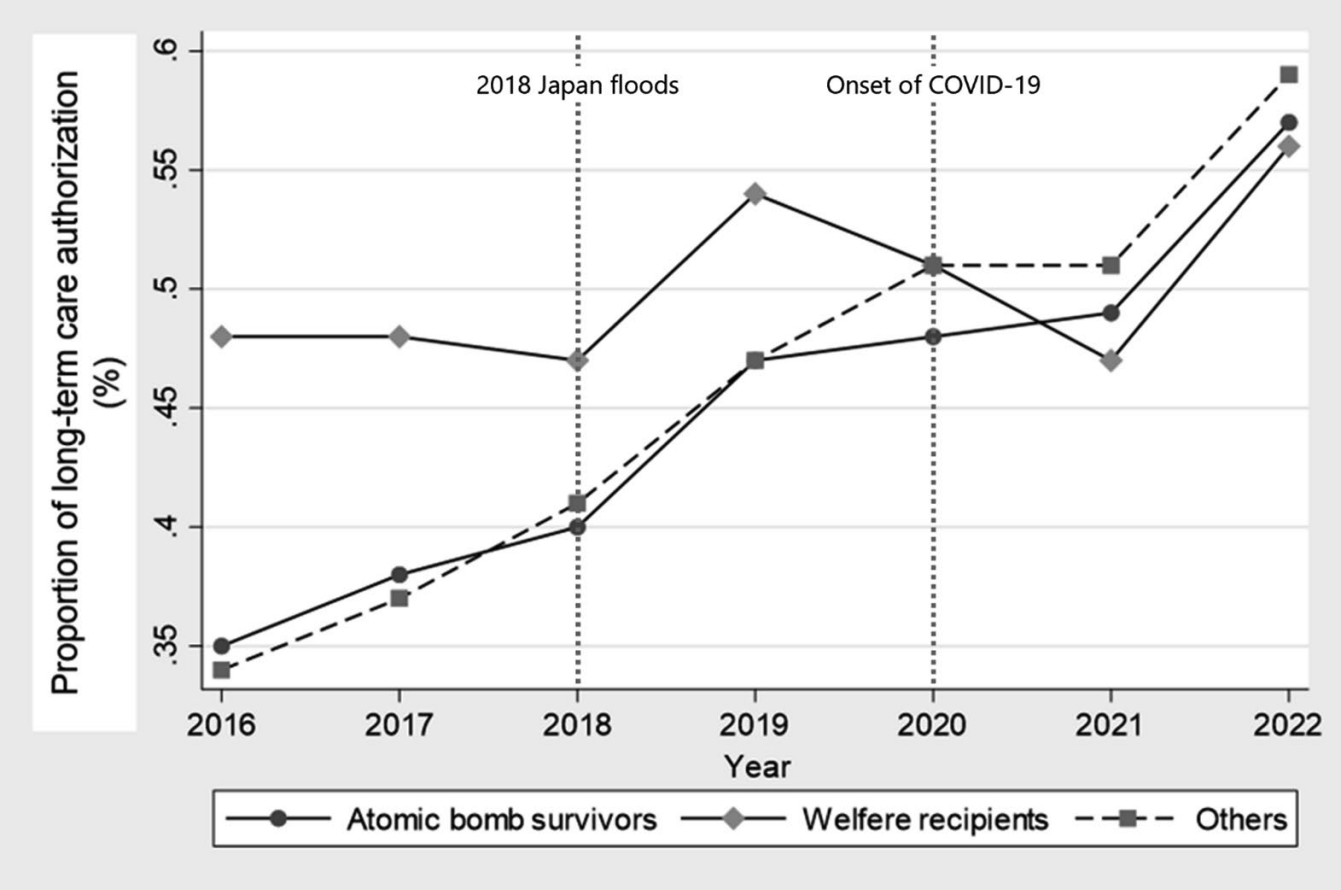
Proportion of long-term care certification. This is the proportion of participants in each group who received care certifications each year. Although ABSs and others showed an increasing trend in care certifications, WRs remained at relatively high levels since the observation period began

Figure 1 shows the proportion of participants in each group who received care certifications each year. Although ABSs and others showed an increasing trend in care certifications, WRs remained at relatively high levels since the observation period began.

Table 2 shows the intervals of care certification before and after the COVID-19 pandemic (2016–2019/2020–2022). The proportion of ABSs with intervals exceeding 1 year before the pandemic and exceeding 2 years after the pandemic was significantly higher than the expected proportion based on the proportion of other participants.

**Table 2:** Number of cases and residual analysis before and after the pandemic for the period of long-term care certification

Year: 2016–2019						
Interval of long-term care certification	Atomic bomb survivors		Welfare recipients		Others	
	n	%	n	%	n	%
- Half year	2,007^−−^	7.3	966^+^	8.9	16,808^+^	8.2
Half year − 1 year	11,335^−−^	41.3	4,398^−−^	40.6	88,113^++^	43.1
1 year − 2 year	9,237^++^	33.7	3,490	32.2	65,394^−−^	32
2 year -	4,866^++^	17.7	1,976^++^	18.2	33,949^−−^	16.6
Year: 2020–2022						
Interval of long-term care certification	Atomic bomb survivors		Welfare recipients		Others	
	n	%	n	%	N	%
- Half year	1,776^−^	8.2	1,038^++^	9.9	17,829	8.7
Half year − 1 year	7,242^−−^	33.3	3,715^−−^	35.5	78,531^++^	38.5
1 year − 2 year	5,118	23.5	2,316^−−^	22.1	48,104^+^	23.6
2 year -	7,602^++^	35	3,406^++^	32.5	59,532^−−^	29.2

−: *p* < 0.05, less than expected value

−−: *p* < 0.001, less than expected value

+: *p* < 0.05, greater than expected value

++: *p* < 0.001, greater than expected value

**Fig. 2:**
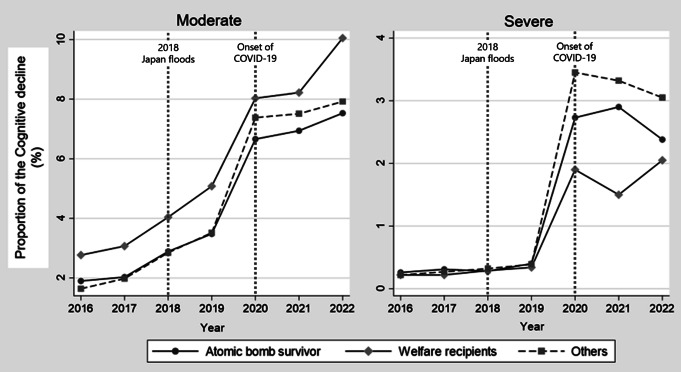
Proportion of cognitive decline to moderate and severe. This is the proportion of participants in each group whose cognitive function deteriorated to at least moderate or severe levels after undergoing care certification in each year. Moderate and severe cognitive decline followed visually parallel trends among all groups before the pandemic. There were no remarkable changes in 2018 (the 2018 Japan floods). Severe cognitive decline increased after 2020

Figure 2 shows the proportion of participants in each group whose cognitive function deteriorated to at least moderate or severe levels after undergoing care certification in each year. Moderate and severe cognitive decline followed visually parallel trends among all groups before the pandemic. There were no remarkable changes in 2018 (the 2018 Japan floods). Severe cognitive decline increased after 2020.

**Fig. 3:**
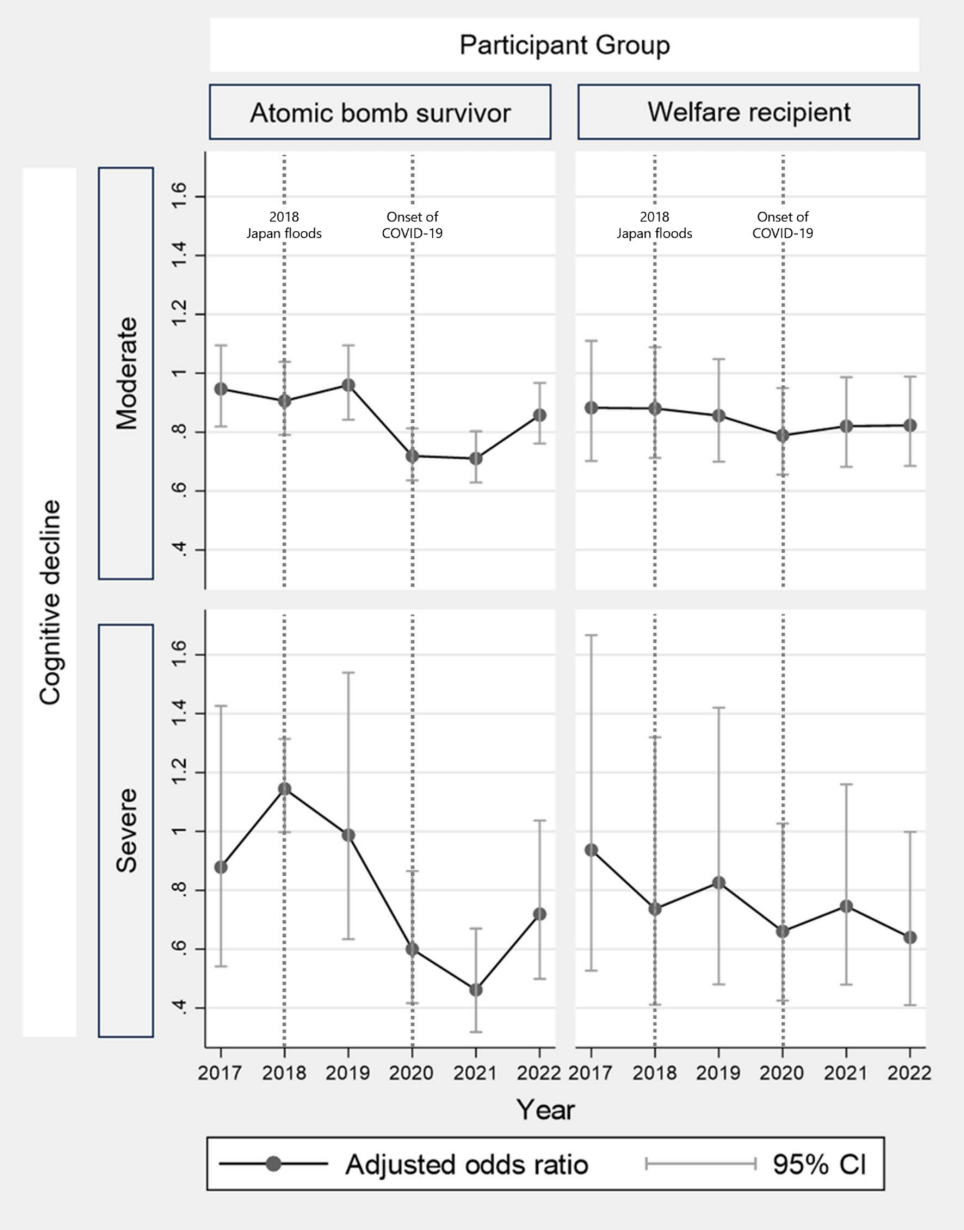
Adjusted odds ratios for cognitive decline among atomic bomb survivors and welfare recipients compared with those of others, using a difference-in-differences analysis. The DID analysis showed no statistical significance for the 2018 Japan floods. The DID results indicated that moderate cognitive decline among ABSs and WRs decreased during the COVID-19 pandemic (2020, 2021, and 2022). Moreover, severe cognitive decline in ABSs decreased after the onset of the COVID-19 pandemic (2020 and 2021)

Figure 3 shows the odds ratios for cognitive decline in ABSs versus others and WRs versus others using DID analysis. The interaction terms between the pre-event years (2016 and 2017) and ABS/WR status were all close to one, indicating that the parallel trends were valid. The DID analysis showed no statistical significance for the 2018 Japan floods. The DID results indicated that moderate cognitive decline among ABSs and WRs decreased during the COVID-19 pandemic (2020, 2021, and 2022). Moreover, severe cognitive decline in ABSs decreased after the onset of the COVID-19 pandemic (2020 and 2021).

## Discussion

Although many older adults experienced cognitive exacerbations during the COVID-19 pandemic, ABSs had a lower risk of cognitive decline than those of non-WRs. However, no significant changes were observed during the 2018 Japan floods.

During the COVID-19 pandemic, cognitive deterioration, particularly severe cognitive decline, increased in all three groups. A regional cohort study in Japan indicated that the cognitive function of patients with preexisting dementia worsened because of the COVID-19 pandemic [22]. Similar trends have been reported in other countries [23]. Despite these trends, ABSs were at a lower risk of cognitive exacerbation compared with that of non-WRs. Because of an aging society, the number of long-term care certifications and longer certification intervals has increased [24]. Long-term care certifications can be renewed without ending the certification period. Therefore, if a person experiences worsening disease conditions or a decline in activities of daily living and requires long-term care services beyond the certified level, they can reapply for care certification. Additionally, the certification period is generally longer for those in stable health conditions [25]. In other words, although the number of care certifications is increasing, the certification period tends to be longer for individuals in stable health conditions. Because many ABSs require care as they age, their stable condition may have contributed to their lower risk of cognitive decline.

The ABSs included in this study survived despite an increased risk of various diseases [26]. It can be reasonably assumed that the majority of ABSs who were at higher risk had already succumbed to cancer, leukemia, and other diseases, as well as factors affecting life expectancy. The surviving ABSs in this study likely had fewer radiation-related diseases and more stable health conditions, making them less susceptible to cognitive decline during the pandemic. Psychosocial factors may have also played a role because of their labeled identity as ABSs. For example, Hiroshima City and Hiroshima Prefecture provide annual medical check-ups for ABSs [27, 28]. Moreover, ABSs have long been exposed to information about their increased disease risk due to radiation exposure. Loneliness and social isolation during the COVID-19 pandemic have been linked to cognitive decline in older adults [29]. It is possible that public support from local governments, social connections among ABSs, and their general tendency to trust others helped mitigate loneliness and social isolation during the COVID-19 pandemic [7]. WRs exhibited a trend similar to ABSs in terms of moderate cognitive decline. Because both groups were exempt from out-of-pocket medical and long-term care expenses, economic barriers to healthcare access during the pandemic may have influenced their cognitive function. However, there is no direct pathological link between radiation exposure in 1945 and the emergence of infectious diseases in 2020–2022. Therefore, the results of this study suggest that biopsychosocial factors, rather than radiation exposure, contributed to the maintenance of cognitive function in ABSs during the COVID-19 pandemic.

However, in 2018, during the Japan floods, ABSs did not exhibit significant changes in cognitive function compared with others. This disaster resulted in approximately 28,000 evacuees [31]. By December 2018, most general evacuation centers had closed, indicating that many victims had returned to normal living conditions within 6 months. By contrast, the COVID-19 pandemic, which began in 2020, had prolonged effects for over 3 years, until legal designations were modified in May 2023 [32]. This difference in duration may have contributed to the lack of disparity in cognitive decline between ABSs and other groups. Additionally, the contrasting nature of the initial responses to these events—group evacuation during the 2018 Japan floods vs. isolation during the COVID-19 pandemic—may have influenced these results. Furthermore, flood victims were not directly identified in this study. Cognitive function declined among older individuals identified as disaster victims [8, 9]. Because ABS victims of the floods could not be specifically identified in the database, this study observed changes in the overall population, weakening the results and preventing the detection of significant changes.

Although the number of cases of moderate cognitive decline increased even before the COVID-19 pandemic, no apparent increasing trend was observed for severe cognitive decline during the same period (Fig. 2). However, after the start of the COVID-19 pandemic, the number of severe cognitive decline cases increased in all groups. In addition to isolation caused by the COVID-19 pandemic, those close to frail older individuals were no longer able to provide informal support to prevent COVID-19 transmission, as the tendency to refrain from meeting separately residing family members increased [34]. The COVID-19 pandemic also increased caregiver burden and caused severe psychological distress in informal caregivers [35]. Therefore, caregivers had to manage LTCI more carefully. The number of direct COVID-19-related deaths in Hiroshima Prefecture from 2020 to 2022 was reported to be 1,373, according to the MHLW [36]. However, this study revealed that the number of individuals whose cognitive function deteriorated to a severe level was nearly 19,436 (ABSs: 799, WRs: 1,060, others: 18,376), approximately 14 times the number of deaths. With the emergence of infectious diseases, the number of older individuals with severe cognitive decline will continue to rise, making it necessary to establish care strategies that incorporate infection prevention even during non-pandemic periods.

This is the first study to assess changes in cognitive function among ABSs using long-term care receipt data from all certified users during the COVID-19 pandemic. Because this was an observational study, it was difficult to match the participant characteristics, including ABS status, WR status, other covariates, unmeasured confounding factors, and cognitive function among the three groups (ABS/WR/Others). Moreover, in a before-and-after study without a control group, it was necessary to adjust for the natural historical trends over time. A DID analysis addresses these two biases if the time-series data of the exposure and control groups meet the common trend assumption prior to an event. Figure 2 shows the parallel trend among the three groups until 2019 visually. The interaction terms between the pre-event years (2016 and 2017) and the ABS/WR status were not statistically significant, indicating that the parallel trends were also valid. Therefore, we could remove the effects of these two biases from the results of this study. Additionally, this study comprehensively examined the cognitive function of ABSs in Hiroshima following the 2018 Japan floods and COVID-19 pandemic. Furthermore, the database used is highly accurate, as it is maintained by the MHLW.

This study had several limitations. First, the database contains limited information on deaths, diseases, hospital visits, treatments, and hospitalizations. Thus, it was not possible to determine which medical conditions contributed to cognitive decline, including COVID-19. To address this limitation, we conducted a DID analysis to adjust for unmeasured bias. Additionally, it is possible that many ABSs lived alone and were less likely to be formally evaluated for cognitive function. However, because this study focused on individuals certified for LTCI services, care providers and managers were able to observe them. Although family-provided information may have been limited, care providers and care managers could still assess major cognitive decline. Finally, although ABSs retain their certification over time, WRs may lose certification depending on their financial situation. Individuals who received WR certification only once during the study period were classified as WRs. Therefore, even if a person was certified for only a short period, they were still registered as a WR. Consequently, the findings may have been diluted compared with an analysis restricted to individuals who consistently required welfare certification. However, since the analysis in this study was conducted to examine trends in comparison with ABSs, this limitation did not significantly impact the study’s objective.

## Conclusions

The COVID-19 pandemic has increased the number of older individuals experiencing cognitive decline, highlighting critical issues that should be considered in future pandemic response strategies. By contrast, ABSs had a reduced risk of cognitive deterioration during the pandemic compared with that of others. A more detailed analysis of ABSs could help identify specific preventive measures against cognitive decline in future pandemics.

## Data Availability

No datasets were generated or analysed during the current study.
